# Full-scale computation for all the thermoelectric property parameters of half-Heusler compounds

**DOI:** 10.1038/srep22778

**Published:** 2016-03-07

**Authors:** A. J. Hong, L. Li, R. He, J. J. Gong, Z. B. Yan, K. F. Wang, J. -M. Liu, Z. F. Ren

**Affiliations:** 1 Laboratory of Solid State Microstructures and Innovation Center of Advanced Microstructures, Nanjing University, Nanjing 210093, China; 2 Department of Physics and TcSUH, University of Houston, Houston, TX 77204, USA

## Abstract

The thermoelectric performance of materials relies substantially on the band structures that determine the electronic and phononic transports, while the transport behaviors compete and counter-act for the power factor *PF* and figure-of-merit *ZT*. These issues make a full-scale computation of the whole set of thermoelectric parameters particularly attractive, while a calculation scheme of the electronic and phononic contributions to thermal conductivity remains yet challenging. In this work, we present a full-scale computation scheme based on the first-principles calculations by choosing a set of doped half-Heusler compounds as examples for illustration. The electronic structure is computed using the WIEN2k code and the carrier relaxation times for electrons and holes are calculated using the Bardeen and Shockley’s deformation potential (DP) theory. The finite-temperature electronic transport is evaluated within the framework of Boltzmann transport theory. In sequence, the density functional perturbation combined with the quasi-harmonic approximation and the Klemens’ equation is implemented for calculating the lattice thermal conductivity of carrier-doped thermoelectric materials such as Ti-doped NbFeSb compounds without losing a generality. The calculated results show good agreement with experimental data. The present methodology represents an effective and powerful approach to calculate the whole set of thermoelectric properties for thermoelectric materials.

At least as much as the energy we used on earth is lost in the form of waste heat^1^. Thermoelectric (TE) power generators that enable the direct conversion from heat to electricity have been studied for a long time, much earlier than the claimed energy crisis^2^,^3^,^4^,^5^,^6^,^7^,^8^,^9^,^10^,^11^,^12^. A good TE material should have high figure of merit *ZT* = (*S*^2^*σ*/*κ*_*tot*_)*T*, where *S*, *σ*, *κ*_*tot*_ (=*κ*_*l*_ + *κ*_e_), *T*, represent the Seebeck coefficient, electrical conductivity, total thermal conductivity, and absolute temperature, and *κ*_*l*_ and *κ*_*e*_ are the lattice and electronic components, respectively. The thermally-driven electrical performance of a TE material is measured by the power factor (*PF* = *S*^2^*σ*), while a high heat-to-electricity conversion efficiency is scaled by *ZT*. Conceptually, in order to possess a large *ZT*, the *PF* must be large and the total thermal conductivity *κ*_*tot*_ should be minimized. Good electrical conduction usually corresponds to high thermal conductivity and a counteracted relationship between the *S* and *σ* is often observed, resulting in the complex relationships between these physical parameters (*S*, *T*, *σ*, *κ*_*l*_, and *κ*_e_). Given this dilemma, an optimization of all these properties so that the largest *PF* and *ZT* can be obtained simultaneously is far beyond fast-track experimental explorations. By the way, technically, a reliable measurement of the *κ*_*tot*_ and evaluation of its two components (*κ*_*l*_, *κ*_*e*_) seem to be tricky and thus the reported data are sometimes authors-dependent. These issues are thus appealing materials computation and property design as a pre-requisite for exploring TE materials for favorable applications. As a result, a theoretical prediction from first-principles calculations and other methods has been of interest for a long time^13^,^14^,^15^,^16^. For example, Yan *et al.*^15^ developed a scheme to calculate the carrier mobility, effective mass, and lattice thermal conductivity related to TE performances, and Sparks *et al.*^16^ proposed an approach of data mining to search for novel TE materials, both of which are of significance. Nevertheless, developing a full-scale computation scheme for the TE properties of a material to guide the experimental search is still appealed.

However, such a scheme remains yet to be found, in particular for carrier-doped TE materials. There exist two major challenges for an accurate calculation of these TE properties. First of all, given reliable knowledge on electronic structure as produced by the *ab-initio* calculations and/or experimental probing using techniques like angle-resolved photoelectron spectroscopy for a TE compound, indeed the semi-classic Boltzmann transport theory can be employed to predict the *σ*, *S*, and *κ*_*e*_. Even though, earlier calculations on these parameters (*σ*, *S*, *κ*_*e*_) utilizing this semi-classic theory relies on a constant relaxation time (*τ*) or non-constant relaxation time obtained from the experimental data^12^,^17^,^18^ which are certainly questionable for many cases. This drawback damages the reliability of the predicted properties. In our study, we use the deformation potential (DP) theory combined with the effective mass approximation to calculate the relaxation time (*τ*) which is no longer treated as a constant. In this method, the effective mass is very important to the calculation of relaxation time (*τ*). The effective carrier masses are often calculated by a fitting of energy bands along the high symmetry lines, which is not accurate. In our work, we calculate the effective carrier masses at all ***k*** points in the first-Brillouin zone, and then obtain the average of the effective masses at all specific energies. For the *p*-type and *n*-type semiconductors, we respectively take the average effective masses at the top of the valence band and the bottom of the conduction band. If the ***k***-point-mesh is dense enough, the effective carrier mass calculation using our method is accurate.

Second, it is known that the lattice thermal conductivity *κ*_*l*_ is mainly determined by the three-phonon processes (corresponding to the intrinsic lattice thermal conductivity denoted by *κ*_*l-in*_), impurity scattering, and boundary scattering. The latter two processes make the calculation particularly difficult at the current stage. On one hand, a prediction of *ZT* is a formidable task because it requires accurate carrier relaxation time for evaluating *σ* and *κ*_*e*_. On the other hand, a first-principles computation of the *κ*_*l*_, especially for doped TE materials would demand computational resource that is too big to access in the material design routine. Along this line, an alternative computation scheme partially free of this difficulty would be favorable. In fact, Bardeen and Shockley proposed in 1950s the deformation potential (DP) theory to interpret the carrier transport in semiconductors where the carriers are mainly scattered by acoustic phonons. Consequently, the density functional perturbation theory (DFPT) combined with the quasi-harmonic approximation (QHA) can be used to calculate the *κ*_*l*_ of a stoichiometric compound. Within the framework of this modified density functional theory, the *κ*_*l*_ of a doped compound can be evaluated by the Klemens’ equation. To this end, a full-scale computation of the whole set of TE parameters for doped TE compounds is thus possible.

In this work, we will demonstrate this scheme by applying it to doped half-Heusler alloys which have received much attention for their good TE performance at the intermediate temperature, less than 900 K. These alloys offer high *PF* and high thermal stability. For instance, the XNiSn (X = Zr, Hf) compounds possess large Seebeck effect (*S* ~ 100–500 μV/K at *T* ~ 300 K) and moderately low electrical resistivity. An optimization by microstructure engineering and doping allows a reproducible *ZT* ~ 1.0 at *T* ~ 900 K^19^,^20^,^21^,^22^,^23^,^24^,^25^,^26^,^27^,^28^,^29^. For MgAgSb-based half-Heusler alloys, a proper optimization allows the *ZT* value as large as ~1.0 at room temperature and ~1.4 at *T* ~ 475 K^30^. In particular, the *p*-type NbFeSb-based half-Heusler alloys have their *ZT*s up to ~1.1 at *T* ~ 1100 K^31^. It is shown that these materials are always doped and experiments revealed the substantial change of thermal conductivity upon varying carrier density (*n*). Therefore, the calculated *κ*_*l*_ from the measured total *κ*_*tot*_using the Wiedemann-Franz relation is much less reliable, which on the contrary enhances the significance of the present work.

Our calculations start from the half-Heusler NbFeSb compound and consider the Ti substitution for Nb as dopant. Since this compound has the face-centered cubic structure with only three atoms in the primitive cell, a calculation of phonon dispersion behavior becomes possible. A description of the scheme and procedure of the full-scale computations is presented in the Methods section below.

## Results

### Electronic structures

**Figure 1:**
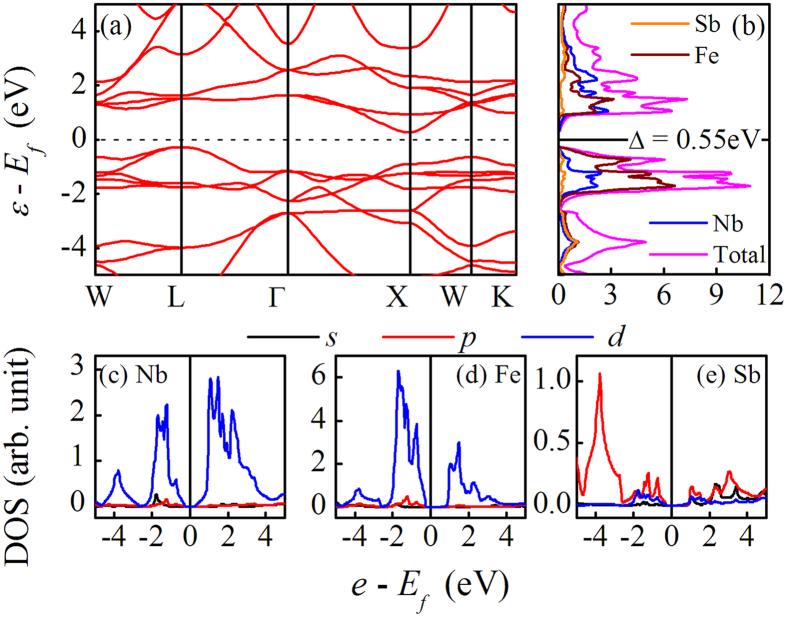
The calculated band structure (**a**) and DOS spectra for Fe atoms, Nb atoms, and Sb atoms, as well as the total DOS (**b**) for NbFeSb compound. The projected DOS for Nb atoms (**c**), Fe atoms (**d**), and Sb atoms (**e**) in NbFeSb compound are plotted too.

The crystal structure of NbFeSb belongs to the #216 space group^31^, as illustrated in Fig. S1 of the supplementary materials. The calculated electronic dispersion relations along the high symmetry lines and thus evaluated density of states (DOS) are shown in Fig. 1(a,b). It is seen that the NbFeSb lattice has the indirect band gap of ~0.55 eV and band degeneracy at the valence band maximum (VBM). The major DOS contributions to the VBM come from the Fe atoms, while almost identical contributions of the Fe and Nb atoms to the DOS near the conduction band minimum (CBM) are identified. For both cases, the contributions from the Sb atom around the VBM and CBM are quite weak. These imply that the Seebeck effect is mainly induced by the Fe and Nb atoms rather than the Sb atoms.

For further illustrating the bonding characteristics, one looks at the bonding peaks between Fe and Nb atoms. The projected DOS profiles for the *s-*, *p-*, *d-*orbitals of these atoms in the energy interval between −5.0 eV and 5.0 eV are presented in Fig. 1(c,d), and the electronic density distribution on the (011) plane is shown in Fig. S2 of the supplementary materials. The *d*-orbitals from the Nb and Fe atoms contribute mostly to the total DOS, while the DOS contribution from the Sb atoms mainly comes from the *p*-orbitals, which shows that there is the *p-d* hybridization between the Sb-Fe pairs and Sb-Nb pairs.

### Electrical transport

**Figure 2:**
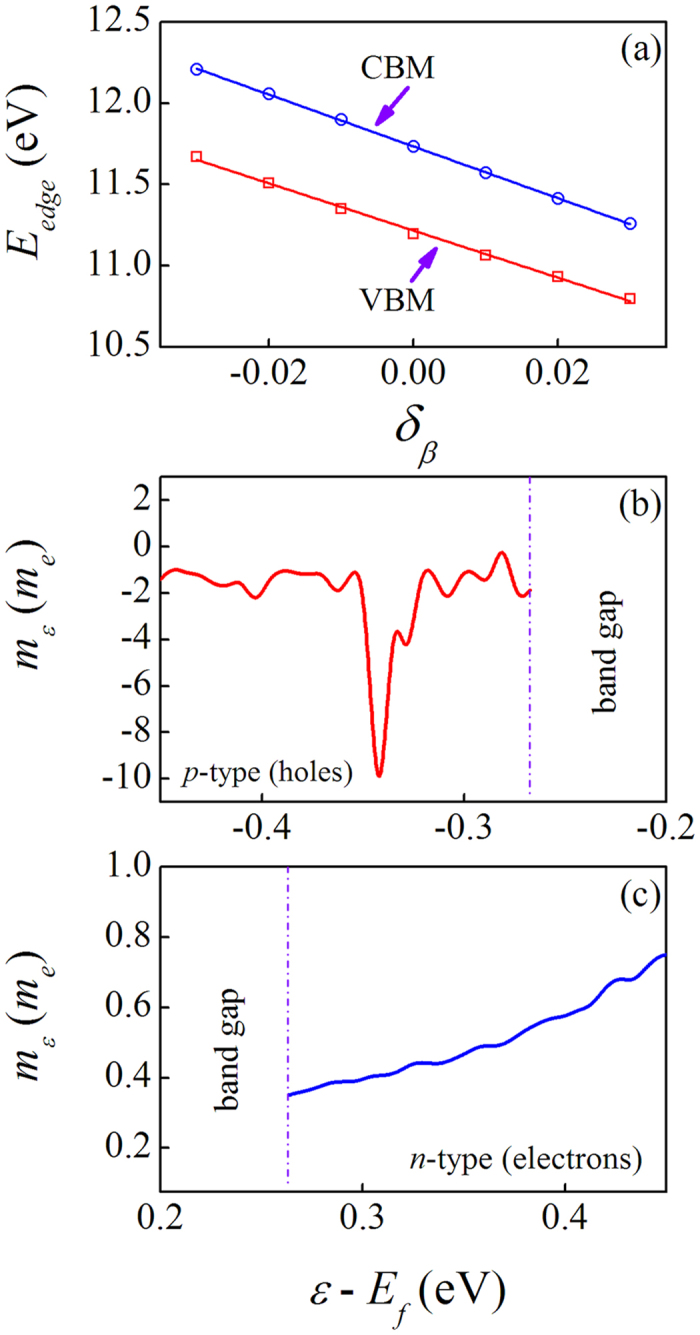
The edge energy shifts (*E*_*edge*_) for the conduction band (CBM) and valence band (VBM) with respect to the lattice dilation along the *a*-axis (**a**) and the energy *ε*-dependent effective masses for holes (**b**) and electrons (**c**). The red lines are the fitting curves.

For stoichiometric NbFeSb compound and its Ti-doped counterparts Nb_1−*x*_Ti_*x*_FeSb, the available physical parameters are shown in Table S1 of the supplementary materials. The calculated elastic constants *c*_*ij*_, bulk modulus *B*, and shear modulus *G*_*H*_ are listed in Table S2 of the supplementary materials. Given these constants and the obtained electronic band structure, the electrical transport behaviors are characterized by parameters (carrier effective mass *m*, carrier mobility *μ*, carrier relaxation time *τ*) for electrons and holes. Figure 2(a) presents the band edge energy (*E*_*edge*_) values at the CBM and VBM for electrons and holes as a function of the uniaxial strain *δ*_*β*_ assigned along the *a*-axis, respectively, exhibiting good linear dependence. Here, the average electrostatic potential^32^ is set as a reference to obtain the absolute band edge shifts. The DP constant *λ*_*β*_ values, as listed in Table 1, are similar for electrons (*λ*_*β*_ = −15.94 eV) and holes (*λ*_*β*_ = −14.51 eV). The calculated *m* at zero temperature, and mobility *μ* and relaxation time*τ* at *T* = 300 K for electrons and holes, are shown in Table 1. It is seen that *τ* = 245.04 fs for electrons is almost 12 times longer than that for the holes, while the mobility for electrons is 1018.46 cm^2^ V^−1^ s^−1^, ~66 times larger than that for the holes. We also investigated the energy (*ε*) - dependent effective carrier mass *m*_*ε*_ in the valence band and conduction band, as shown in Fig. 2(b) where *m*_*e*_ is the electron mass. In the valence band, the effective mass is positive, indicating the hole-like behavior of the energy band due to the concave curvatures, while the carriers in the conduction band show electron-like behaviors. The effective mass near the VBM is ~1.87 *m*_*e*_, much heavier than ~0.35 *m*_*e*_ near the CBM. This difference results in the huge discrepancies of the carrier relaxation time and mobility for holes and electrons.

**Figure 3:**
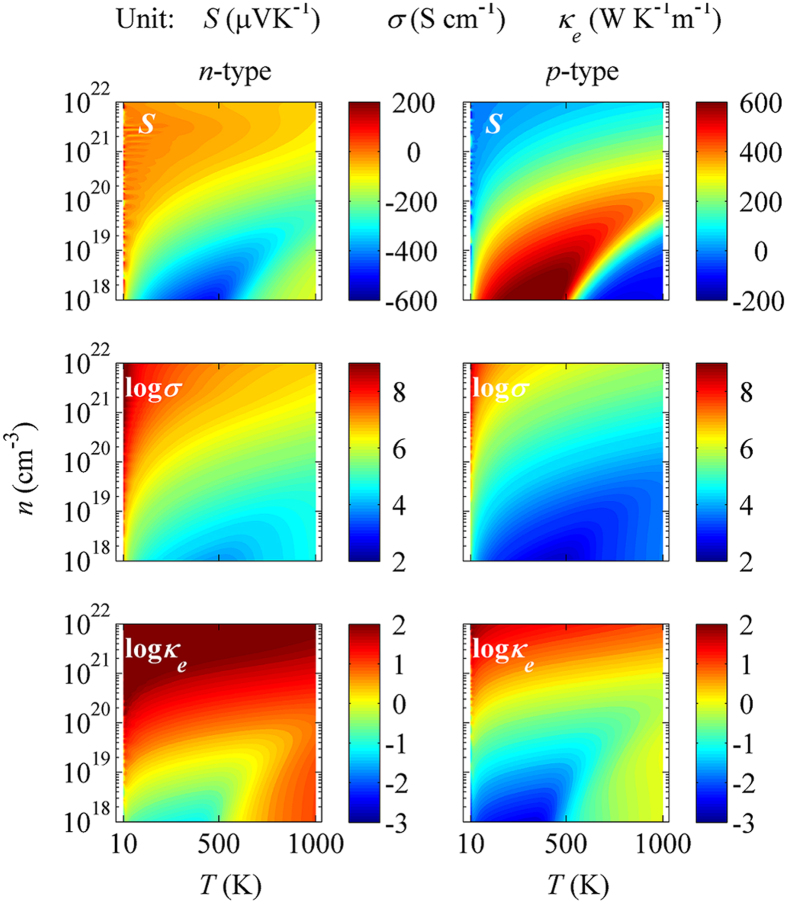
The calculated *S* (top row), log*σ* (middle row), and log*κ*_*e*_ (bottom row) on the (*T*, *n*) plane for carrier-doped NbFeSb. Left panel: *n*-type and right panel: *p*-type.

We present extensive calculations on these TE properties (*σ*, *S*, *κ*_*e*_), given either the *p*-type doping or the *n*-type doping with a broad range of carrier density *n*. The results are summarized in Fig. 3 where the parameters in the (*T*, *n*) plane are plotted on the right column for hole-doping and on the left column for the electron-doping. In the overall sense, these parameters all show non-trivial variations. First, the counter-acting relationship between *S* and *σ* (or *κ*_*e*_) can be established for both doping cases. The large |*S*| appears in the region of low *n* and intermediate *T*, but both *σ* and *κ*_*e*_ are low in this region. Second, in the low *n* region, one observes only insignificant variation of *σ* over the *T*-range covered here, but *κ*_*e*_ exhibits remarkable increasing with increasing *T*. This remarkable increasing is much more than the linear *T*-dependence as predicted by the Wiedemann-Franz law *κ*_*e*_ = *LσT* where *L* = (1.49~2.45) × 10^−8^ W K^−2^ is the Lorenz constant. Third, these non-trivial dependences of (*σ*, *S*, *κ*_*e*_) on (*T*, *n*) suggest that an optimized design in terms of the TE performance by carrier doping strategy is necessary.

### Lattice thermal transport

**Figure 4:**
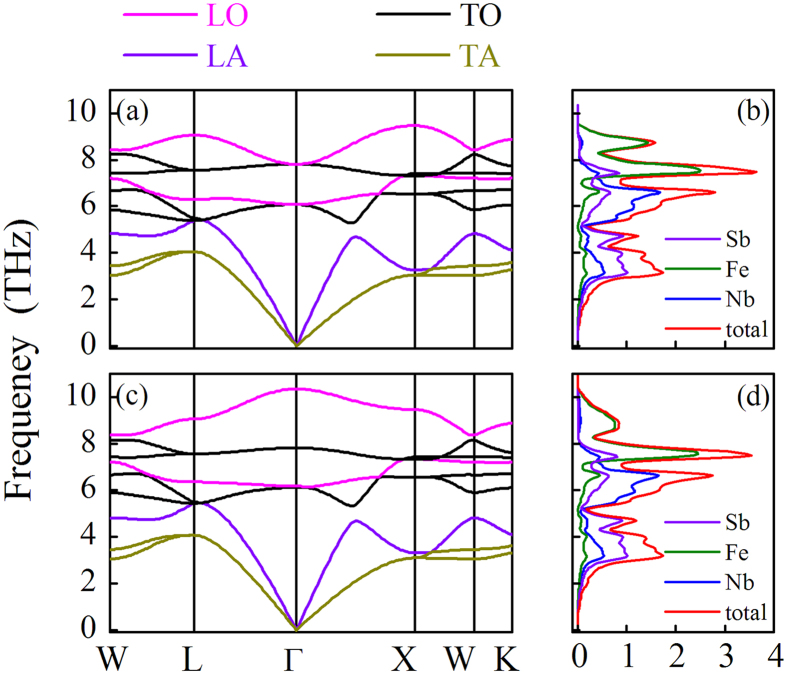
The calculated phonon spectra (**a**) and phonon DOS (**b**) without the LO-TO splitting for NbFeSb compound. The corresponding spectra and DOS with the LO-TO splitting are plotted in (**c,d**) respectively.

The more critical issue is to evaluate accurately parameter *κ*_*l-in*_ (intrinsic thermal conductivity) for NbFeSb compound and *κ*_*l*_ for the doped Nb_1−*x*_Ti_*x*_FeSb. First, the phonon band structures are shown in Fig. 4(a,c), respectively, for FeNbSb lattice without and with non-analytical term correction along the representative symmetry lines within the first Brillouin zone of the primitive cell with three atoms. The three atoms give rise to nine phonon branches, *i.e*., one longitudinal acoustic (LA) mode, two transverse acoustic (TA) modes, two longitudinal optical (LO) modes, and four transverse optical (TO) modes. The two TA modes along the Γ-L and Γ-X directions are two-fold degenerate. The acoustic and optical branches overlap near the L point.

It is known that the electric dipoles caused by displacement of charges of long-wavelength LO modes can lead to internal electric field. The phonon frequencies for the LO modes at the Γ point will be up-shifted by this induced electric field. Thus, the LO-TO splitting is an important parameter to evaluate the strength of ionicity. A comparison of Fig. 4(a,c) shows the clear non-vanishing LO-TO splitting at the zone-center of the Brillouin zone, which implies the existence of the ionic bonding. It is seen from Fig. 4(a) that the frequencies of the two triply degenerate optical phonons at the Γ point are 6.07 THz and 7.80 THz, respectively. Surely, one of the two triply degenerate optical phonons is split into two-fold degenerate and single-fold degenerate optical phonons when the LO-TO splitting effect is taken into account. Subsequently, we can obtain the phonon density of states by taking the LO–TO splitting into account, as shown in Fig. 4(d) evaluated from Fig. 4(c). It is seen that the low-frequency branches up to 5.6 THz are mainly from the Sb atomic vibrations, while the frequency branches between 5.6 THz and 7.1 THz are mainly from the Nb atomic vibrations. The Fe atomic vibrations contribute to the high-frequency branches above 7.1 THz.

Based on the above consideration, one can calculate a set of parameters for evaluating the thermal conductivity, including the Grüneisen parameter *γ*, isometric heat capacity *C*_*V*_, Debye temperature Θ_*D*_, and intrinsic lattice thermal conductivity *κ*_*l-in*_ as a function of *T* for NbFbSb lattice. The results are summarized in Fig. S3 of the supplementary materials. The values of *γ*, *C*_*V*_, and Θ_*D*_ are 1.69, 69.03 J K^−1^ mol^−1^, and 384.90 K at *T* = 300 K. For *T* > 300 K, the calculated *κ*_*l-in*_(*T*) curve is plotted in Fig. S3(d), which decreases monotonously with increasing *T*, from ~22.0 W K^−1^ m^−1^ at *T* = 300 K to ~6.0 W K^−1^ m^−1^ at *T* = 1000 K. Given the *κ*_*l-in*_(*T*) data, the *κ*_*l*_ for Nb_1−*x*_Ti_*x*_FeSb with different doping level *x* is calculated by Eq. (20) in the Methods section. For this calculation, relationship *x*_*per*_ = *x*/3 = *nV/*3Δ*Z* should be satisfied, where *V* is the volume of unit cell and Δ*Z* is the valence difference between the master atom (Nb) and substituting atom (Ti). The values of these parameters are given in Table S1 of the supplementary materials.

To this stage, with *κ*_*tot*_ = *κ*_*l*_ + *κ*_*e*_, *PF* = *σ*·*S*^2^, and *ZT* = *σ*·*S*^2^·*T*/*κ*_*tot*_, one reaches a full-scale computational scheme for the whole set of TE parameters for a TE compound or doped TE alloy. In the following section, we apply this scheme to the doped compounds Nb_1−*x*_Ti_*x*_FeSb with different *x* for TE performance optimization.

### Optimization of TE performance in Nb_1−x_Ti_x_FeSb

**Figure 5:**
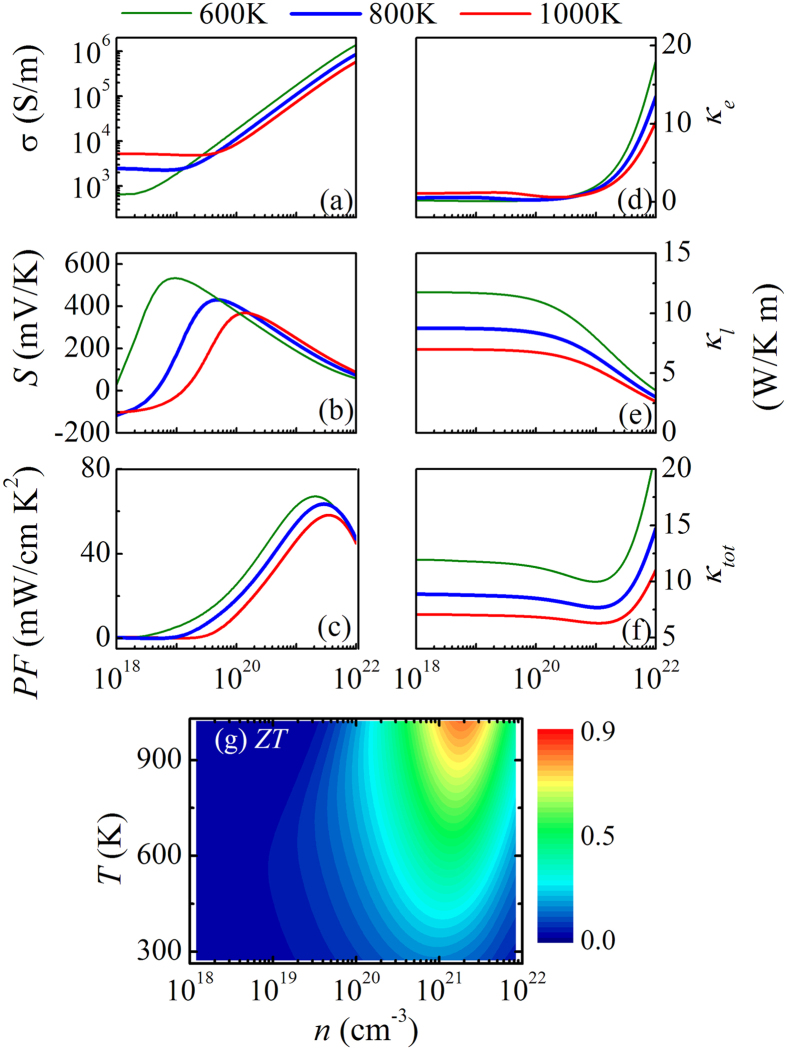
The calculated electrical conductivity *σ*(*n*) (**a**), Seebeck coefficient *S*(*n*) (**b**), power factor *PF*(*n*) (**c**), electronic thermal conductivity *κ*_*e*_(*n*) (**d**), lattice thermal conductivity *κ*_*l*_(*n*) (**e**), and total thermal conductivity *κ*_*tot*_(*n*) (**f**) at three temperatures *T* = 600 K, 800 K, and 1000 K, respectively, for the *p*-type Nb_1−*x*_Ti_*x*_FeSb alloys. The evaluated figure of merit *ZT* on the (*T*, *n*) plane is plotted in (**g**).

We have performed extensive calculations on the whole set of TE parameters for a series of *p*-type Ti doped Nb_1−x_Ti_x_FeSb compounds, considering the nominal substitution of Nb^5 + ^ $ by Ti^4 + ^. Several representative sets of data on these parameters as a function of *n* at *T* = 600, 800, and 1000 K are summarized in Fig. 5(a–f). It is noted here that the calculated results represent a spatial averaging over the three major axes [100], [010], and [001]. Given the cubic lattice structure, these parameters along the three major axes are nearly identical with one and another. It is seen that a linear dependence of log*σ* on *n* is identified in the intermediate and high *n* ranges. A linear dependence of log*σ* on *n* is identified in the intermediate and high *n* ranges. The *σ*(*n*) is sensitive to *T* but show weak dependence on *n* in the low *n* ranges. The *S*(*n*) first increases and then decreases with increasing *n*, exhibiting a single-peaked pattern. With increasing *T*, the peak height of *S*(*n*) decreases, nevertheless, the peak location increases. As expected, the *S*(*n*) and *σ*(*n*) exhibit the opposite dependences, and both are insensitive to *T* in the high *n* range. The *PF*(*n*) first increases and then decreases with increasing *n*, similar to the *S*(*n*) curves. The peak height becomes low and the peak location shifts to the high *n* range with increasing *T*.

More interested are the calculated *κ*_*e*_(*n*), *κ*_*l*_(*n*), and *κ*_*tot*_(*n*). The *κ*_*e*_(*n*) remains low in the low-*n* range (<10^21^ cm^−3^), beyond which a rapid increase of *κ*_*e*_(*n*) is identified. The *κ*_*l*_(*n*), instead, shows the saturated plateaus in the low-*n* range and then falls rapidly in the high-*n* range due to electron-phonon scattering. Due to the different *n*-dependences and similar magnitudes of *κ*_*e*_(*n*) and *κ*_*l*_(*n*), the *κ*_*tot*_(*n*) exhibits strong *n*- and *T*-dependences: less sensitive to *n* in the low-*n* range but highly sensitive to *n* in the high-*n* range. Conclusively, we summarize the *ZT*(*n*, *T*) contour in Fig. 5(g). While the *ZT* is quite low over most of the region unfortunately, a large *ZT* ~ 0.86 is obtained at the optimal condition (*n* ~ 1.45 × 10^21^ cm^−3^ and *T* ~ 1000 K). The *ZT* values are ~0.39 and ~0.62 at *T* = 600 K and 800 K, given *n* ~ 1.45 × 10^21^ cm^−3^.

### Comparison with experiments

**Figure 6:**
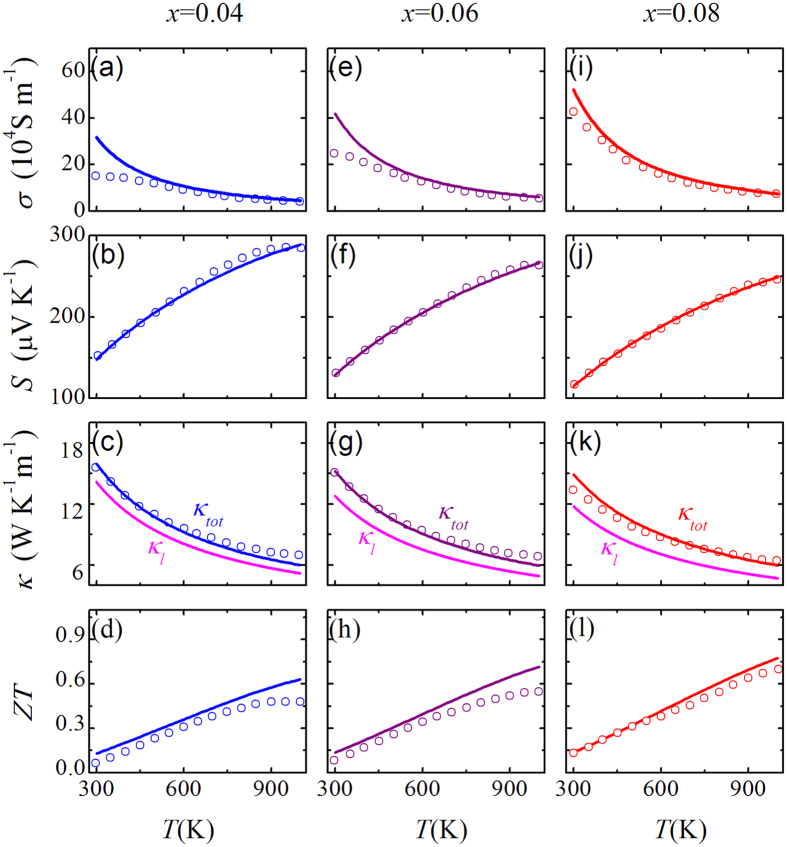
The evaluated electrical conductivity *σ*(*T*), Seebeck coefficient *S*(*T*), otal thermal conductivity *κ*_*tot*_(*T*), and *ZT*(*T*) for Nb_1−x_Ti_x_FeSb alloys at *x* = 0.04 (**a–d**), *x* = 0.06 (**e–h**), *x* = 0.08 (**i–l**), respectively. The solid lines are the calculated results and the dots are measured data extracted from ref. 31.

Finally, we compare our calculated data quantitatively with measured data on Nb_1−x_Ti_x_FeSb^31^, noting that no data for *x* = 0.0 itself are available. The measured *S*, *σ*, and *κ*_*tot*_ data for polycrystalline samples at *x* = 0.04, 0.06 0.08, are taken from ref. 31 with the *n* of ~6 × 10^20^ cm^−3^, ~9 × 10^20^ cm^−3^, and ~12 × 10^20^ cm^−3^, respectively, giving the ratio *x*/*n* of ~0.67 × 10^−22^ cm^3^. By inputting these parameters we obtain *σ*(*T*), *S*(*T*), *κ*_*l*_(*T*), *κ*_*tot*_(*T*), and *ZT*(*T*) data as plotted in Fig. 6(a–l), where the dots are the measured data and the solid lines are from calculations. Experimentally, the *κ*_*l*_(*T*) data are extracted from the *κ*_*tot*_(*T*) data by excluding the *κ*_*e*_(*T*) estimated by the Wiedemann-Franz relation. Even so, the calculated *κ*_*l*_(*T*) is still consistent well with the extracted ones.

For all the three doped cases, the calculated *S*(*T*) and *κ*_*tot*_(*T*) match with measured data nicely over the whole *T*-range. It indicates that our computational scheme works well for predicting the *S* and *κ*_*l*_ and suggests that the imposed approximations with this scheme don’t induce remarkable uncertainties to the two parameters at least. However, the measured *σ* is lower than the calculated values in the low *T*-range. This discrepancy can be partially ascribed to the microstructural defects like grain boundaries, impurities, and other defects *etc*, scattering the carriers and decreasing the electrical conductivity particularly in the low *T*-range where the carrier scattering by these defects and impurities is important. In the high *T*-range, the carrier scattering from optical phonons may be neglected, and this may partially explain the slightly higher calculated *σ* than the measured one. The measured thermal conductivity is bigger than the calculated in the high *T*-range which is due to that the contribution from optical phonon to thermal conductivity is neglected in the calculation. Consequently, the difference between the calculated *κ*(*T*) and measured ones makes the calculated *ZT* values larger than measured ones, as shown in Fig. 6(d,h,l). It is noted that recent studies^15^,^33^ incorporated a minimum optical contribution (a constant) to the lattice thermal conductivity at high *T* and obtained good agreement with measured lattice thermal conductivity for materials with big number of atoms in the unit cell. In this study, the NbFeSb compound has only three atoms in the unit cell, a minimum optical contribution (a constant) to the lattice thermal conductivity is negligible. In addition, the NbFeSb compound is believed to be polar to some extent. Although the carrier relaxation time *τ* is less affected by the polarization field, the induced contribution may be one of the origins for the difference in *σ*(*T*).

## Discussion

The present computational scheme has been demonstrated for the whole set of TE properties on half-Heusler NbFeSb compound. This scheme combines efficiently the multi-scale computation techniques, but the imposed several approximations may introduce substantial discrepencies in some cases such as synthesis-dependent polycrystalline samples. They are deserved for additional discussions.

First, the TE parameters depend substantially on the microstructures and associated defects. The carrier scattering from electron-phonon interaction, polarization electric field, impurities, defects, and grain boundaries, *etc*., may be important. However, only the electron-phonon interaction is considered in the present scheme. Substantial uncertainties may arise and in particular the electrical conductivity does show deviations from measured results. Based on the constant relaxation time approximation, it is believed that the Seebeck effect is independent of carrier relaxation *τ* and determined by the Fermi level *Ef* (or *n*) and electronic structure, allowing much better consistency of the calculated values with measured ones.

Second, the calculated parameters are obtained by spatially averaging the data on single crystal along the three main axes. Both the lattice symmetry and anisotropy make this averaging inaccurate. Fortunately, the NbFeSb compound has cubic structure, reasonably insuring the applicability of the present computational scheme. Surely, this scheme can be easily extended to those systems of high lattice symmetry and low polar nature, and the extension is straightforward, noting that the Debye approximation which ignores the optical phonons should be modified for calculating the anisotropic *κ*_*l*_.

Third and surprisingly, it is interesting to note that the calculated *κ*_*tot*_ data are also in good agreement with measured ones for the polycrystalline samples synthesized by ball-milling method offering fine grain size. These fine-structured features, not considered in the present scheme, may add additional scattering processes to the phonon transport. The good agreement seems to suggest the dominant role of the long-wavelength phonons in the lattice thermal conductivity, and therefore these multi-scale microstructural features may work for further reducing the lattice thermal conductivity.

Finally, for a general sense, we have to remind that a capricious utilization of the present computation scheme to predict a potential TE material should be anyhow cautious. We list several of these considerations: (1) electronic and phononic structures are in fact dependent of carrier doping/element substitution; (2) mixing of covalent bonding and ionic bonding makes the relation between substitution level (*x*, *x*_*per*_) and carrier density (*n*) complicated; (3) the applicability of the Slack’s equation and the Klemen’s equation should be concerned. Indeed, an optimal prediction/design of a TE material is a collection of many physical parameters which are inter-related, and a reliable design remains to be challenging.

## Methods

### Electrical transport

For the electronic structure, we employed the density functional theory (DFT) with full-potential linearized augmented plane-wave (LAPW) method implemented in WIEN2k code^34^ which can offer enough dense *k*-mesh to make transport coefficients converge. The exchange and correlation interactions are described using generalized gradient approximation (GGA) and Perdew-Burk-Ernzerhof (PBE) functional^35^. For practical computation, the muffin-tin radii can be set as 2.0 *a.u.* for all the atoms and the plane-wave cut-off is defined by *R*_*MT*_·*K*_*n*_ = 8.0, while an energy threshold of −8.0Ry is usually used in order to separate core and valence states. It is noted that we don’t include the spin-related contribution for transport properties of NbFeSb compound. It is usually assumed that the magnetic order has a slight impact on the electro-transport above 300 K.

Given the electronic structure, the electrical transport parameters at finite *T* are obtained by solving the Boltzmann transport equation^36^. In our calculation, the calculated transport coefficients are well converged using a shifted 43 × 43 × 43 *k* mesh. The original *k*-mesh is interpolated onto a mesh 5 times as dense. The electrical conductivity tensors *σ*_*αβ*_ and electronic thermal conductivity tensors 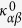 at non-zero electric current are given^36^:


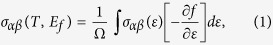



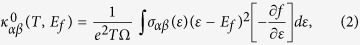


where subscripts *α* and *β* stand for the two axis directions in the momentum space (or corresponding real space), parameters *e*, Ω, *ε*, and *f* represent the electron charge, unit cell volume, carrier energy, and Fermi distribution function, respectively, and *E*_*f*_ is the Fermi energy. The *σ*_*αβ*_ can also be expressed as a function of *ε*:


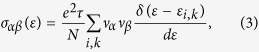


where *τ*, *N*, and *ν*_*α*_ (*ν*_*β*_) are the carrier relaxation time, number of sampled *k*-points, and carrier group velocity along the *α*(*β*) direction. Subscripts *i* and *k* are the band index and the wave-vector. In the standard procedure, Seebeck coefficient *S* and electronic thermal conductivity tensors *κ*_*αβ*_ at zero electric current can be given by^36^:


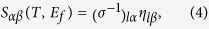



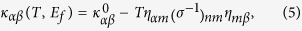


where subscripts (*l*, *m*, *n*) stand also for the axis directions in the momentum space, while the main axes (*a*, *b*, *c*) in real space are parallel to those axes in the momentum space. The coefficient tensor *η*_*αβ*_ at arbitrary two directions (*α, β*) are written as:


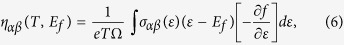


For an approximately isotropic system like NbFeSb compound, the *σ*, *S*, and *κ*_*e*_ can be directly evaluated from *σ* = (*σ*_*11*_ + *σ*_*22*_ + *σ*_*33*_)/3, *S* = (*S*_*11*_ + *S*_*22*_ 0 *S*_*33*_)/3, and *κ*_*e*_ = (*κ*_*11*_ + *κ*_*22*_ + *κ*_*33*_)/3, which all are the functions of *T* and *E*_*f*_ (or *n*).

Based on the constant relaxation time approximation, the Seebeck effect is determined mainly by the band structure and roughly irrelevant with relation time *τ*. Nevertheless, both the *σ* and *κ*_*tot*_ are strongly dependent on *τ*. An evaluation of *τ* alone from the *ab-initio* data is inaccurate since it depends on phonon scattering and polarization electric field if the lattice is polar, and also on impurity and defects. For simplified consideration, the influences from impurity and defects can be safely neglected in high *T* range, and we only need to consider the phonon scattering. The effective mass approximation based on the DP theory is used to evaluate the *τ*. Accordingly, the carrier relaxation time *τ* and mobility *μ* defined specifically at the conduction (valence) band edge for a three-dimensional lattice can be expressed as^37^:


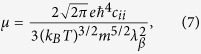



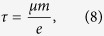


where *k*_*B*_ is the Boltzmann constant, *c*_*ii*_ is the lattice elastic constant (*i* = 1, 2, 3), and *λ*_*β*_ is the DP constant defined as:


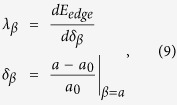


where *δ*_*β*_ is the uniaxial strain along the *β* direction and this strain is defined by the variation of lattice constant *a* with respect to equilibrium lattice constant *a*_*0*_ along the *β* direction. In Eqs (7) and (8), *m* is the isotropic effective carrier mass at the conduction (valence) band edge, which can be calculated by:


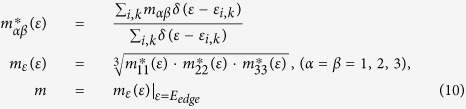


where 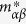 is the energy-dependent effective mass tensor and tensor *m*_*αβ*_ is given by


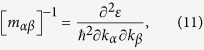


with 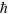 the Planck constant. It is noted that the effective carrier mass 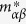 is obtained by averaging the *m*_*αβ*_ over the whole momentum space.

Finally, the carrier density *n* is obtained via:


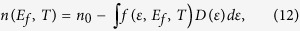


where *n*_*0*_ is the valence electron number and *D*(*ε*) is the total density of states (DOS) as a function of *ε*, evaluated from the electronic structure. There is the one-to-one correspondence between *n* and *E*_*f*_ at a given *T*.

### Elastic properties

An evaluation of the *τ* and *μ* needs the *c*_*ij*_ of a material and they are calculated using the strain method (energy approach) in the WIEN2k code. For this specific computation, the special points sampling integration over the Brillouin zone is realized using the Monkhorst-Pack method with 10000 special *k*-points meshes. For cubic lattice such as NbFeSb compound, there are three independent elastic constants, *i.e*., *c*_11_, *c*_12_, and *c*_44_, whose evaluation requires three independent homogeneous distortion modes. The first is the orthorhombic distortion mode satisfying the volume-conservation, the second is to change the lattice parameter in the *a*-axis, given the conserved cubic symmetry, and the third is the monoclinic distortion mode with varying lattice parameter along the *a*-axis satisfying the volume-conservation rule too. According to the Voigt’s and Reuss’s approximations, the shear modulus can be expressed as^38^:


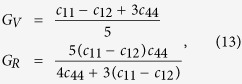


Hill *et al. λ* proposed the mean arithmetic value of *G*_*V*_ and *G*_*R*_ to reflect the real properties of a material:


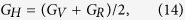


The bulk modulus *B* is defined as:


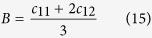


### Lattice thermal conductivity

Now one can calculate the *κ*_*l*_. We first discuss the *κ*_*l-in*_. The DFPT^39^ combined with the QHA is employed. The DFPT is a combination of the standard DFT with a linear electron density response, which is highly favorable for calculating the phonon frequencies over the Brillouin zone efficiently. In the specific operation, we specified the volume changes in 3%, 2%, 1%, 0%, −1%, −2%, −3% for the QHA. The Slack’s equation which applies at high *T* > Θ_*D*_ yields^40^:


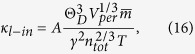


where *A* is a dimensionless collection of physical constants (*A* ~ 3.1 × 10^−6^), Θ_*D*_ the Debye temperature, *V*_*per*_ the volume per atom, *n*_*tot*_ the number of atoms in the primitive unit cell, 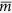 the average mass of all the atoms in the crystal, *γ* is the Grüneisen parameter defined as^41^:


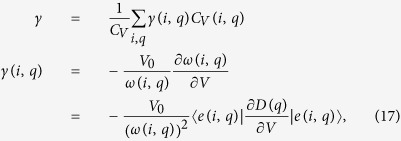


where *i* and *q* are the band index and the phonon wave vector, *C*_*V*_ and *C*_*V*_(*i*, *q*) are the isometric heat capacity and mode heat capacity, respectively, *γ*(*i*, *q*) is the mode Grüneisen parameter^42^, *V*_0_ is the equilibrium volume, *ω*(*i*, *q*) is the phonon frequency of the *i*-th branch at wave vector *q*, *D*(*q*) is the dynamical matrix, and *e*(*i, q*) is the eigenvector. The *C*_*V*_(*i*,*q*) and then *C*_*V*_ are calculated from the phonon dispersions


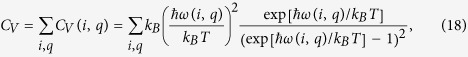


and in general, the *C*_*V*_ and *γ* are for all the phonon modes including the acoustic and optical modes.

On the other hand, the Θ_*D*_ is evaluated by calculating the Θ_*D*_/*T* truncated expression of the isometric heat capacity *C*_*V*_^43^:


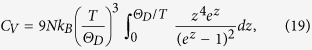


where symbol *z* stands for *ћω/k*_*B*_*T.*

To this stage, the lattice thermal conductivity *κ*_*l*_ for a carrier-doped TE compound can be obtained directly by the Klemens’ equation^44^,^45^,^46^:


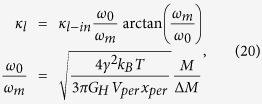


where *ω*_*m*_ and *ω*_*0*_ are the Debye frequency and cut-off frequency, respectively, *M* is the mass of the master atom to be substituted, Δ*M* is the difference in mass between the master atom (Nb here) and substituting atom (Ti here), and *x*_*per*_ is the dopant number fraction per unit cell. The carrier density *n* can be written as *n* ~ 3*x*_*per*_ Δ*Z*/*V* where *V* is the volume of unit cell and Δ*Z* is the difference in valence between the master atom and substituting atom. The negative and positive Δ*Z* values represent respectively the *n-*type and *p*-type carriers. For Nb_1−*x*_Ti_*x*_FeSb, one can have *x*_*per*_ = *x*/3. It is noted that the ratio *ω*_*0*_/*ω*_*m*_ can be negative upon a negative Δ*M*, but the *κ*_*l*_ remains positive.

To this end, the *κ*_*l*_ can be self-consistently calculated once the phonon spectrum is available. Specifically, the phonon spectrum calculation is performed using the VASP (Vienna *ab initio* simulation package) code^47^,^48^. A supercell of 2 × 2 × 2 primitive cell containing three atoms is considered, which consists of a total of 24 atoms. The first-principles calculations based on the DFPT are performed using the VASP code under the generalized gradient approximation (GGA) Perdew-Becke-Erzenhof (PBE) functional. A 6 × 6 × 6 mesh for the first Brillouin-zone sampling and 500 eV for cutoff of the plane-wave basis set are used. It is noted that the longitude optical (LO)-transverse optical (TO) splitting effect is taken into account in the phonon calculation. With the obtained phonon spectrum, the *κ*_*l-in*_ and *κ*_*l*_ are obtained accordingly using the Slack’s equation and the Klemens’ equation.

## Additional Information

**How to cite this article**: Hong, A. J. *et al.* Full-scale computation for all the thermoelectric property parameters of half-Heusler compounds. *Sci. Rep.* **6**, 22778; doi: 10.1038/srep22778 (2016).

## Supplementary Material

Supplementary Information

## Figures and Tables

**Table 1:** The calculated *DP* constant *λ*_*β*_, carrier effective mass *m* at zero temperature, and carrier mobility *μ* and relaxation time *τ* at *T* = 300 K for electrons and holes.

Carrier type	*λ*_*β*_ (eV)	*m* (*m*_*e*_)	*μ* (cm^2^V^−1^s^−1^)	*τ* (fs)
electrons	−15.94	1.87	1018.46	245.04
holes	−14.51	0.35	18.63	19.84
