# Supplementary material for: Full-scale computation for all the thermoelectric property parameters of half-Heusler compounds
Source: Sci Rep. 2016 Mar 7;6:22778. doi: 10.1038/srep22778 (PMC4780035; doi:10.1038/srep22778)
Supplement: Supplementary Information [file srep22778-s1.pdf]

SUPPLEMENTARY MATERIALS

Full-scale computation for all the thermoelectric property parameters of half-Heusler compounds

A. J. Hong[1], L. Li[1], R. He[2], J. J. Gong[1], Z. B. Yan[1], K. F. Wang[1], J. –M. Liu[1], & Z. F. Ren[2]

[1]*Laboratory of Solid State Microstructures and Innovation Center of Advanced Microstructures, Nanjing University, Nanjing 210093, China*

[2]*Department of Physics and TcSUH, University of Houston, Houston, TX 77204, USA*

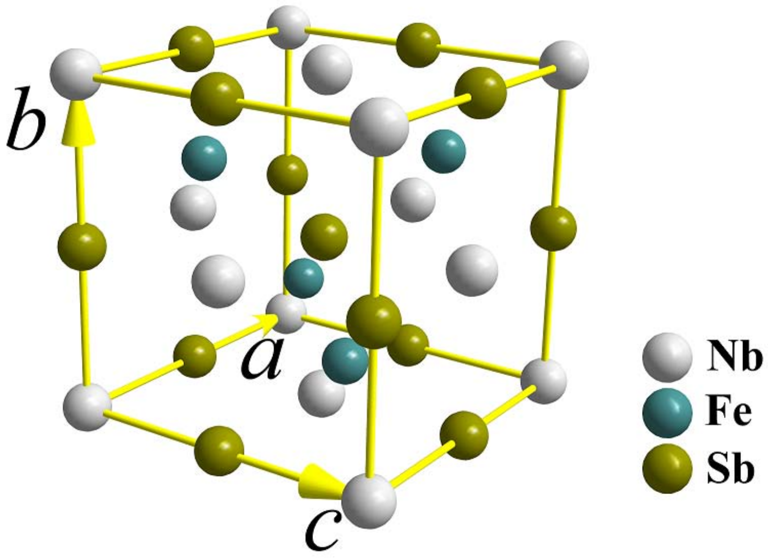

Figure S1. (Color online) A schematic drawing of lattice structure of NbFeSb compound.

The crystal structure of NbFeSb can be viewed as a rocksalt lattice filled with Nb and Sb atoms (occupying the Wyckoff positions 4a and 4b). The Fe atoms fill half of the tetrahedral interstitial sites (either the Wyckoff position 4c or 4d). In our calculation, the #216 space group is maintained and we start from experimentally determined lattice constant a_0 = 5.9587 Å for volume optimization, where all the atoms in the conventional cell are allowed to relax sufficiently till the minimization of the forces less than 0.5 mRy/a.u. The optimized lattice constant is a_0 = 5.9575 Å. The electronic structure calculation is then performed on this optimized lattice.

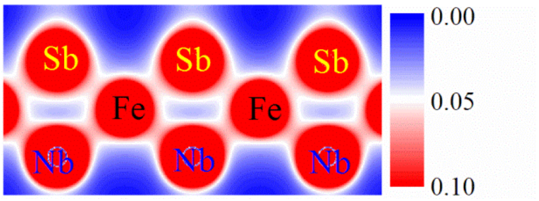

Figure S2. (Color online) The calculated valence electron charge density on the (011) plane for NbFeSb compound.

The electron cloud has remarkable deformation and overlaps around and between all atoms, again implying the strong covalent bonding in the NbFeSb compound.

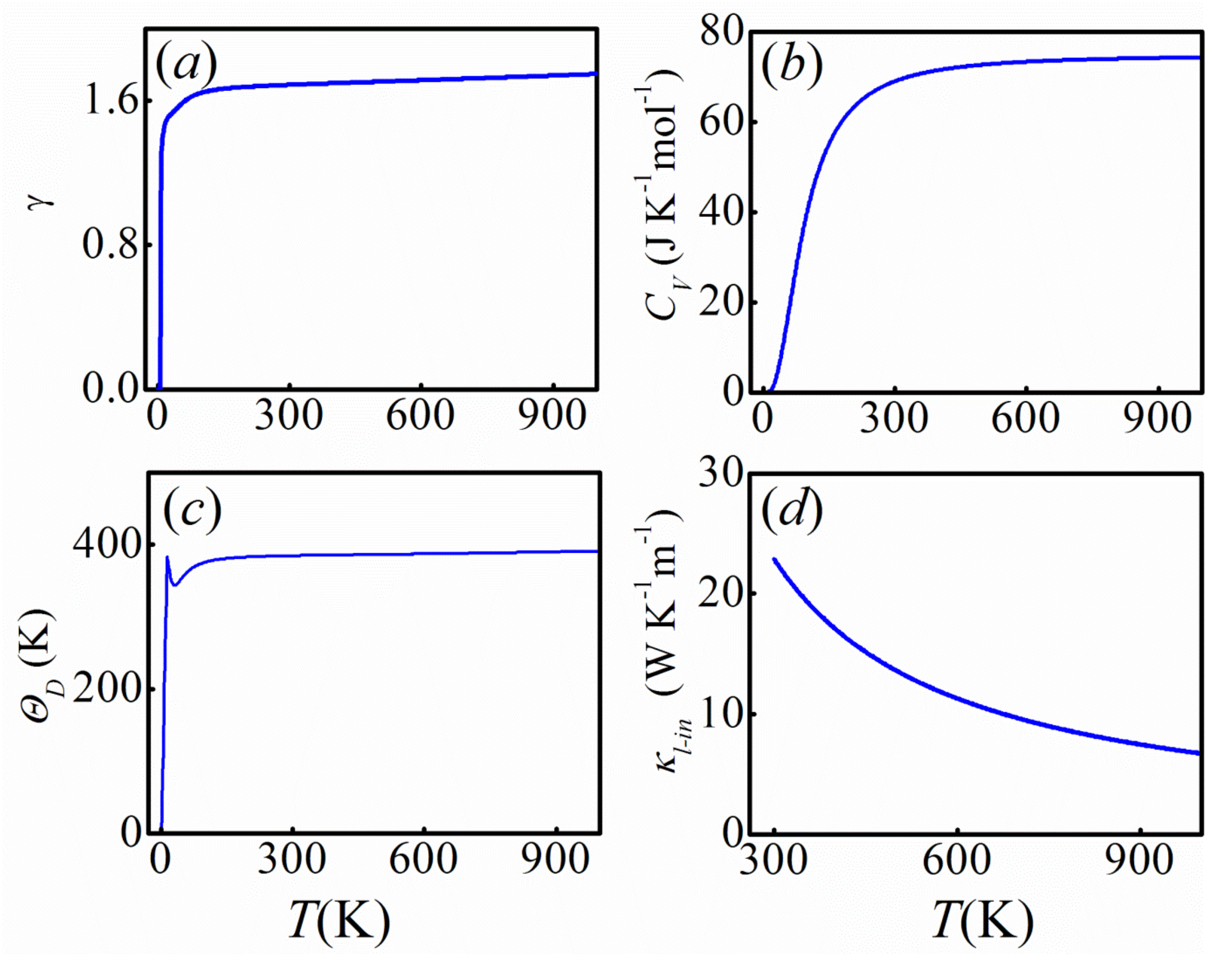

Figure S3. (Color online) The calculated Grüneisen parameter γ (a), isometric heat capacity C_V (b), Debye temperature Θ_D (c), and intrinsic lattice thermal conductivity $\kappa_{l\text{-}in}$ (d) as a function of T for NbFbSb compound.

Table S1. the volume per atom V_{per}, the number of atoms in the unit cell n_{tot}, the average mass $\overline{m}$, the mass of the master atom to be substituted M, the difference in mass between the master atom (Nb here) and substituting atom (Ti here) ΔM, the volume of unit cell V, the difference in valence between the master atom and substituting atom ΔZ for NbFeSb and $Nb_{1-x}Ti_xFeSb$.

V_{per} (Å^3)	n_{tot}	$\overline{m}$ (u)	M (u)	ΔM (u)	V (Å^3)	ΔZ (Nb^{5+}/Ti^{4+})
17.62	3.00	90.17	92.91	45.04	53	1.0

Table S2. The calculated elastic constants c_{ii}, bulk modulus B and shear modulus G_H for NbFeSb compound (unit: GPa).

c_{11}	c_{12}	c_{44}	B	G_H
306.18	92.34	43.61	163.62	63.04
